# Long-Term Cd Exposure Alters the Metabolite Profile in Stem Tissue of *Medicago sativa*

**DOI:** 10.3390/cells9122707

**Published:** 2020-12-17

**Authors:** Annelie Gutsch, Sophie Hendrix, Gea Guerriero, Jenny Renaut, Stanley Lutts, Saleh Alseekh, Alisdair R. Fernie, Jean-Francois Hausman, Jaco Vangronsveld, Ann Cuypers, Kjell Sergeant

**Affiliations:** 1 GreenTech Innovation Center, Environmental Research and Innovation Department, Luxembourg Institute of Science and Technology, 5, Avenue des Hauts-Fourneaux, 4362 Esch-sur-Alzette, Luxembourg; annelie.gutsch@ulb.ac.be (A.G.); gea.guerriero@list.lu (G.G.); jenny.renaut@list.lu (J.R.); jean-francois.hausman@list.lu (J.-F.H.); 2 Centre for Environmental Sciences, Campus Diepenbeek, Hasselt University, Agoralaan Building D, 3590 Diepenbeek, Belgium; shendrix@uni-bonn.de (S.H.); jaco.vangronsveld@uhasselt.be (J.V.); ann.cuypers@uhasselt.be (A.C.); 3 Institute of Crop Science and Resource Conservation (INRES), University of Bonn, Friedrich-Ebert-Allee 144, 53113 Bonn, Germany; 4 Groupe de Recherche en Physiologie Végétale, Earth and Life Institute—Agronomy, Université Catholique de Louvain, 5, Place Croix du Sud, 1348 Louvain-la-Neuve, Belgium; stanley.lutts@uclouvain.be; 5 Max-Planck-Institute of Plant Molecular Physiology, Am Mühlenberg 1, 14476 Potsdam, Germany; alseekh@mpimp-golm.mpg.de (S.A.); Fernie@mpimp-golm.mpg.de (A.R.F.); 6 Centre of Plant Systems Biology and Biotechnology, 4000 Plovdiv, Bulgaria

**Keywords:** *Medicago sativa*, cadmium, primary metabolites, secondary metabolites, flavonoids, environmental stress, acclimation

## Abstract

As a common pollutant, cadmium (Cd) is one of the most toxic heavy metals accumulating in agricultural soils through anthropogenic activities. The uptake of Cd by plants is the main entry route into the human food chain, whilst in plants it elicits oxidative stress by unbalancing the cellular redox status. *Medicago sativa* was subjected to chronic Cd stress for five months. Targeted and untargeted metabolic analyses were performed. Long-term Cd exposure altered the amino acid composition with levels of asparagine, histidine and proline decreasing in stems but increasing in leaves. This suggests tissue-specific metabolic stress responses, which are often not considered in environmental studies focused on leaves. In stem tissue, profiles of secondary metabolites were clearly separated between control and Cd-exposed plants. Fifty-one secondary metabolites were identified that changed significantly upon Cd exposure, of which the majority are (iso)flavonoid conjugates. Cadmium exposure stimulated the phenylpropanoid pathway that led to the accumulation of secondary metabolites in stems rather than cell wall lignification. Those metabolites are antioxidants mitigating oxidative stress and preventing cellular damage. By an adequate adjustment of its metabolic composition, *M. sativa* reaches a new steady state, which enables the plant to acclimate under chronic Cd stress.

## 1. Introduction

Heavy metal pollution, being a stressor for plants, is a great concern of the industrial time. Therefore, cadmium (Cd) is a common pollutant found in the soil of industrial sites, which can be taken up by the plant root system and enter the human food chain [1]. Cadmium toxicity in plants causes reduced growth, leaf chlorosis, imbalanced water household and impaired photosynthesis. Cadmium elicits oxidative stress in plant cells by interfering with the activity of various enzymes involved in antioxidative defense [2], misbalancing production of antioxidants such as glutathione (GSH) [3] and disrupting neutralization of reactive oxygen species (ROS) [4,5,6,7]. Enhanced ROS levels lead to DNA damage, lipid peroxidation and protein modification, which interferes with their activity [8,9,10]. Plants have developed several endogenous mechanisms to counteract toxic effects such as the synthesis of metabolites [11].

Metabolites are the biological active compounds in plant cells and a result of the integration of gene expression and protein activity [12]. As such, metabolites reflect the molecular phenotype better than genes or proteins alone and are important to characterize biological processes [13,14]. It is estimated that the number of different metabolites in the plant kingdom ranges between 200,000 and 1,000,000, but it can also just be a few thousands for a single species (approximately 5000 metabolites in *Arabidopsis thaliana*) [15]. Plant metabolites are commonly classified into primary metabolites, secondary metabolites and plant hormones. While primary metabolites such as amino acids (AAs) and polyamines (PAs) are ubiquitous and required for growth and development, the abundance of secondary metabolites is regulated by plant development and environmental factors [16,17]. The latter are classified according to their chemical structure in major groups such as terpenes, alkaloids and phenolics [18]. Phenolic compounds are a remarkably diverse group of secondary metabolites and include lignins, lignans and tannins but also flavones and isoflavones [19]. The distinction into primary metabolites, secondary metabolites and plant hormones became common definitions in plant biology. However, recent studied blur this strict functional separation as secondary metabolites can have regulatory functions like primary metabolites and furthermore reintegrate into primary plant metabolism [20].

One attempt to study primary and secondary metabolites in plants is that of metabolomics, a fast-developing high-throughput approach, which utilizes targeted metabolite quantification or untargeted metabolite profiling [21,22]. Gas chromatography–mass spectrometry, capillary electrophoresis, nuclear magnetic resonance spectroscopy (NMR) and liquid chromatography (LC–MS) are commonly used techniques [23,24]. But the immense variability in chemical structure and properties of metabolites makes it nearly impossible to identify them all. However, recent advances in ultra-high-performance liquid chromatography (UHPLC–MS) have expended the sensitivity, resolution and throughput of this technique for metabolite analysis [25,26]. Metabolomics is frequently used to study plant stress responses to biotic and abiotic stress in order to decipher which molecules are important during stress responses and tolerance acquisition [27,28,29]. The profile of primary metabolites such as AAs was analyzed by ^1^H NMR and GC–MS in response to different heavy metals and revealed a specific metabolic profile in response to the applied stress, which implied a vital role of AAs during stress adaptation [30,31,32]. HPLC and LC–MS measurements were utilized to investigate changes in the profile of phenolic compounds in response to heavy metal stress [33,34,35]. It was demonstrated that heavy metal stress increases the total content of phenols and the application of different heavy metals alters the phenolic profile [36]. Phenolic compounds can act as potential antioxidants by scavenging free radicals and/or act as metal chelators [37,38,39]. The capacity of flavones to bind metals was recently demonstrated in plant extracts [40] and metal chelating properties of flavones were reported with regard to the resistance to aluminium toxicity in maize [41].

The globally important forage crop *Medicago sativa* was and is mainly used for feeding livestock [42]. It is an excellent source of protein and dietary fibers, providing a variety of micronutrients and carbohydrates [43]. The plant leaves have a high protein content, which fits the needs of the feed market. Its less digestible stems represent more than 50% of the produced biomass and are rich in cell wall material. Therefore, *M. sativa* stem tissue is often used in research to study fundamental processes taking place at the cell wall [44,45]. It can be used for industrial purposes such as bioethanol production [46,47] increasing the economic value of the plant. As a rich source of secondary metabolites, *M. sativa* is studied for its bioactive compounds and their application in industry and agriculture [37,48].

In previous studies, the response of *M. sativa* to an environmentally realistic Cd concentration (10 mg kg^−1^ soil) [49] has been investigated focusing on the cell wall [50,51] with a specific emphasis on the stems. Despite representing 50% of the harvestable biomass of *M. sativa*, few studies have specifically focused on the impact of environmental constraints on stems. Long-term Cd exposure induced changes in the gene expression of enzymes in the phenylpropanoid pathway as well as ethylene biosynthesis in stem tissue [52]. Ethylene induces the phenylpropanoid pathway, which provides the precursor molecules for monolignol biosynthesis and lignin formation. Lignin deposition in the cell wall is commonly found in response to Cd exposure [9,53,54]. However, the lignin content did not increase in the stem cell wall as a consequence of Cd exposure [52], although increased cell-wall-located peroxidase abundance [50] and phenylpropanoid pathway induction [52] would have suggested that. Both monolignols and flavonoids derive from the phenylpropanoid pathway (Pawlak-Sprada et al., 2011a, 2011b). Here, targeted and untargeted metabolic approaches were applied using different metabolomics platforms (GC–MS and UPLC–MS) to investigate whether there is a shunt of the shared precursor molecules towards the (iso)flavonoid pathway instead of lignin biosynthesis.

## 2. Materials and Methods

### 2.1. Plant Growth and Sampling

*Medicago sativa* L. (cultivar Giulia) plants were exposed to 10 mg Cd kg^−1^ soil (spiked as CdSO_4_) in a long-term experiment, which represents realistic Cd concentrations found in upper soil [49]. Experimental conditions and sampling were previously described in detail [50]. Metabolite analysis was carried out using the same samples. Briefly, plants were potted in 12 times 12 pots for each condition and grown for 5 months with an intermediate cutting as in agricultural practice. Plants were mainly kept in the greenhouse. Neither temperature nor photoperiod were controlled during the experiment and no fertilizer was applied. Five biological replicates for each condition were taken, with a pool of stem tissue from 24 pots corresponding to one biological replicate. Stems were separated from leaves, the first as well as last two internodes removed and subsequently frozen in liquid nitrogen. Samples were ground in liquid nitrogen and kept at −80 °C till further use. Additionally, the Cd content was determined in all organs: Leaves: 21.54 µg g^−1^ dry weight; Stems: 40.09 µg g^−1^ dry weight; Roots: 169.91 µg g^−1^ dry weight, indicating a significant accumulation of Cd in the different tissues (Cd content in respective control samples: Leaves: 0.31 µg g^−1^ dry weight; Stems: 0.24 µg g^−1^ dry weight; Roots: 2.36 µg g^−1^ dry weight) [50].

### 2.2. Measurement of Glutathione in Stem Tissue

Glutathione (GSH) analysis was adopted from a previously published protocol [55]. All extraction steps were performed on ice. Five replicates of each condition were used for statistical analysis. Approximately 120 mg fresh weight (FW) of deep-frozen ground stem tissue was extracted with 200 mM HCl using a mortar and pestle. After centrifugation (16,000× *g*, 10 min, 4 °C), 350 µL of the supernatant was transferred to a new tube and the pH of each extract was adjusted to 4.5 with 200 mM NaOH. The GSH assay relies on the reduction of 5,5-dithiobis(2-nitro-benzoic acid) (DTNB) to trinitrobenzol (TNB) via GSH that is oxidized to GSSG. The latter is recycled via glutathione reductase (GR). Glutathione reductase (Sigma-Aldrich, Darmstadt, Germany) was suspended in 200 mM NaH_2_PO_4_-EDTA to a concentration of 20 U mL^−1^ and kept in aliquots of 500 µL. For each measurement series, a fresh aliquot was used. To measure only the oxidized glutathione disulphide (GSSG) content, each extract was treated with 2-vinylpyridine to complex free GSH. Extracts were incubated at 20 °C for 30 min and centrifuged (16,000× *g*, 10 min, 4 °C) to eliminate complexed GSH. Total GSH content (GSSG and GSH) was measured in the non-treated extract. Measurements were performed in triplicates in 96-well plates. For the total GSH content, 10 µL of each extract was pipetted into plate wells containing 200 mM NaH_2_PO_4_, 10 mM EDTA, 10 mM NADH and 12 mM DTNB. The reaction was started by adding GR and monitored at 412 nm. A GSH standard curve between 0 and 1000 pmol was generated on the same plate. The GSSG was measured in a similar way using 40 µL of the treated extract and with a standard curve ranging between 0 and 400 pmol.

### 2.3. Quantification of Primary Metabolites

#### 2.3.1. HPLC-FLD Analysis of PAs in Stems

Polyamines (PAs) were extracted from 200 mg ground stem tissues with 500 μL HClO_4_ 4% (*v*/*v*) under vigorous vortexing, then kept at 4 °C for 1 h and subsequently centrifuged at 13,000× *g* at 4 °C for 20 min. The obtained pellet was re-extracted with 500 µL HClO_4_ 4% (*v*/*v*). 1,7-diaminoheptane (5 mg L^−1^) was used as an internal standard. For fluorescence detection, PAs were derivatized by dansylation [56] and the dried extract was dissolved in 1 mL methanol and filtered through 0.45 µm microfilters (Chromafil PES-45/15, Macherey-Nagel, Düren, Germany). A total of 5 µL of the sample was injected on a Nucleodur C18 Pyramid column (125 × 4.6 mm internal diameter; 5 μm particle size) (Macherey-Nagel) at 40 °C. Samples were analyzed by high-performance liquid chromatography coupled with a fluorescence detector (HPLC-FLD) from Shimadzu, equipped with a solvent delivery unit LC-20AT, an SIL-HTc autosampler and an RF-20A Fluorescence Detector (Shimadzu, ‘S Hertogenbosch, The Netherlands). Excitation wavelength was set at 340 nm and the emission wavelength was set at 510 nm. The mobile phase consisted of water (eluent A) and acetonitrile (ACN) (eluent B) with a flow rate of 1.0 mL min^−1^. The gradient program was 40% B to 91% B (20 min), 91% B to 100% B (2 min), 100% B (8 min), 100% B to 40% B (1 min), and column equilibration was at 40% B during 4 min. Six-point calibration curves ranging from 3.125 to 100 µM with custom-made external standard solutions and an internal standard (1,7-diaminoheptane) were used to quantify free PAs. Calibration of the system was confirmed every ten injections by using a check standard solution. Internal standard gave information about the recovery of the extraction and derivatization during the evaluation of PAs content.

#### 2.3.2. UHPLC–DAD Analysis of Amino Acids in Stems

Approximately 50 mg of ground stem tissue were freeze dried prior to extraction. Extraction was done with 400 μL methanol containing 10 mM beta-aminobutyric acid (BABA) as internal standard under vigorous vortexing and subsequent shaking (1400 rpm, 15 min). Two hundred μL of chloroform were added, samples were shaken (1400 rpm, 15 min), 400 μL of H_2_O were added and samples were vortexed and centrifuged (12,000× *g*, 5 min). A total of 600 μL of the supernatant was collected. A 50 μL sample aliquot was first dried using a vacuum centrifuge and resuspended in 50 μL H_2_O prior to analysis. The derivatisation of samples and AA standards was done using the AccQ-Tag Ultra Derivatization Kit (Waters, Milford, MA, USA). In total, 5 μL of samples and 5 μL of the AA standards were mixed separately with 35 μL AccQ-Tag borate buffer. To these mixtures, 10 μL AccQTag reagent (Waters AccQ-Tag Ultra Derivatization Kit) were added, samples were vortexed, spun down and incubated for 10 min at 55 °C. UHPLC–DAD (ultra-high performance liquid chromatography–diode array detector) was performed on an Acquity UPLC system (Waters) equipped with an Acquity tunable UV detector. The used column was Acquity UPLC BEH C18 column (2.1 × 100 mm, 1.7 μm particle size, Waters). The flow rate was 0.7 mL min^−1^ and the column temperature was kept at 55 °C. The injection volume was 3 µL and the detection wavelength was set at 260 nm. Two eluents were used: (A) AccQ Tag eluent; (B) ACN. The following gradient elution was used: 0–0.54 min, 0.1% B; 6.5 min, 9.1% B; 8.50 min, 21.2% B; 8.90–9.50 min, 59.6% B; 9.60–10.10 min, 0.1% B.

#### 2.3.3. GC–MS Analysis of Primary Metabolites in Leaves

Ground *Medicago sativa* leaf tissue was extracted as described previously [57]. Briefly, 50 mg of plant tissues was extracted by adding 700 μL 100% methanol. A total of 700 μL from the extraction liquid was taken and transferred into a new reaction tube, followed by adding 375 μL CHCl_3_ and 750 μL water. After centrifugation, 150 µL of supernatant was vacuum dried, and the residue derivatized for 120 min at 37 °C (40 µL of 20 mg mL^−1^ methoxyamine hydrochloride in pyridine) followed by a 30 min treatment at 37 °C with 70 µL of *N*-Methyl-*N*-(trimethylsilyl)trifluoracetamide. The gas chromatography–mass spectrometry (GC–MS) system used was a gas chromatograph coupled to a time-of-flight mass spectrometer (Pegasus HT TOF–MS, Leco, Saint Joseph, MI, USA). Gas chromatography was performed on a 30 m DB-35 column. The used carrier gas was helium at a constant flow rate of 2 mL s^−1^. Samples were injected by an auto sampler Gerstel Multi Purpose system at an injection temperature of 230 °C. The transfer line and ion source were set to 250 °C. The initial temperature of the oven (85 °C) increased at a rate of 15 °C min^−1^ up to a final temperature of 360 °C. Mass spectra were recorded at 20 scans s^−1^ with m z^−1^ 70–600 scanning range after a solvent delay of 180 s. Chromatograms and mass spectra were evaluated by using Chroma TOF 4.5 (Leco) and TagFinder v4.2 software [57,58].

### 2.4. Quantification of Secondary Metabolites

#### 2.4.1. Extraction

Ground stem tissue samples (150 mg) from *M. sativa* were freeze dried and extractions were done with 1790 µL methanol:water (4:1, *v*/*v*). A total of 10 µL of 4-methylumbelliferone (500 µg mL^−1^) was added to the sample as an extraction internal standard. Samples were homogenized using a vortex (30 s) and shaken for 4 h at room temperature to extract the metabolites. Samples were again vortexed (30 s) followed by centrifugation (20,000 × *g* for 30 min at 4 °C). The supernatant was collected and dried using a vacuum centrifuge. Dried extracts were solubilized in 1460 µL methanol:water (5:95, *v*/*v*) and filtered through a syringe filter (0.2 µm, PTFE Millex-LG; Merck KGaA, Darmstadt, Germany).

#### 2.4.2. UHPLC–MS Analysis

Extracts were analyzed with a Waters Acquity UPLC system coupled to a high-resolution time-of-flight mass spectrometer (TripleTOF 5600+; AB Sciex, Foster City, CA, USA). Samples were analyzed in positive and negative ion mode and each 5 µL aliquot was analyzed in two technical replicates. A reverse-phase Acquity UPLC BEH C18 column (2.1 × 100 mm, 1.7 μm particle size, Waters) was utilized with 0.1% formic acid in water (A) and 0.1% formic acid in ACN (B) as eluents at the following gradient: 0 min, 1% B; 4 min, 1% B; 16 min, 5% B; 35 min, 40% B; 45 min, 100% B; 50 min, 100% B; 53 min, 1% B; 60 min, 1% B at a flow of 0.5 mL min^−1^ and 50 °C as the column temperature. An electrospray ionization (ESI) source was used to ionize compounds with the following parameters for the positive and negative mode: source temperature, 650 °C; ion spray voltage of 4.5 and −4.5 kV, respectively; curtain gas (nitrogen) of 30; nebulizer gas (air) of 55; turbine gas (air) of 50. Precursor charge state selection was set at 1. Survey scans were acquired in 175 ms for information-dependent acquisition (IDA in high sensitivity mode), and product ion scans of the 10 most intense peaks were collected when a threshold of 100 counts per s was reached. The total cycle time was fixed at 2.25 s. Each MS1 scan is the sum of four time bins at a pulser frequency value of 16.4 kHz. A sweeping collision energy setting of 15 eV in positive and −15 eV in negative mode was applied for collision-induced dissociation. The declustering potential was set at 60 and −60 eV in positive and negative mode, respectively. Dynamic exclusion was set for 8 s after 2 occurrences. For MS1, full HR–MS spectra between 100 and 1300 mass-to-charge ratio (m z^−1^) were recorded. MS2 scans were recorded between 25 and 1300 m z^−1^.

#### 2.4.3. Data Processing

Data were processed and analyzed using Progenesis QI (v2.3, Nonlinear Dynamics, Newcastle upon Tyne, UK). Each UPLC–MS run was imported as an ion-intensity map and runs were aligned in the retention time direction. A corporate run representing the compounds in all samples was used for peak picking and was compared to all the runs, to ensure that the same ions are detected in every run. Data were normalized according to total ion intensity. Only features with MS2 data, and both fold-change ≥ 1.5 and *p*-value ≤ 0.05 in two technical replicates were considered for identification. Metabolites were identified by their accurate masses using an in-house database as well as CHEBI 3-star (https://www.ebi.ac.uk/chebi), MassBank, PubChem and the NIST MS/MS database. The output was reviewed with PeakView (v 1.2.0.3, AB SCIEX), Metlin (https://metlin.scripps.edu/index.php) and PubChem databases (https://pubchem.ncbi.nlm.nih.gov) as well as literature data for structure elucidation.

### 2.5. Gene Expression Analyses

The RNAqueous™ Kit (Life Technologies, Carlsbad, CA, USA) was used according to the manufacturer’s instructions for RNA extraction from five biological replicates. The RNA was purified by precipitation with 3 M sodium acetate and 100% isopropanol, subsequently washed with 70% ethanol and resuspended in RNase-free water. The RNA was quantified with a NanoDrop^®®^ ND-1000 spectrophotometer (Thermo Fischer Scientific, Braunschweig, Germany) (A260/280 and A260/230 ratio between 1.9 and 2.5) and a Bioanalyzer (Agilent Life Science, Heverlee, Belgium). The RNAs displayed an RIN value between 8.7 and 9.0. Reverse transcription was carried out with the ProtoScript II Reverse Transcriptase (NEB, Massachusetts, USA) following the manufacturer’s instruction. Sequences for the genes of interest were obtained by searching the Alfalfa Gene Index and Expression Atlas Database (http://plantgrn.noble.org/AGED/index.jsp). Specific primer pairs were designed with Primer3Plus (www.bioinformatics.nl/cgi-bin/primer3plus/primer3plus.cgi) and validated using OligoAnalyzer 3.1 (https://eu.idtdna.com/calc/analyzer) (Table S1). Real-time quantitative PCR (qPCR) runs were performed with the Takyon SYBR Green low ROX (Eurogentec, Seraing, Belgium) in 384 well plates on a ViiA7 Real-Time PCR system (Applied Biosystems). Minimum information for the publication of qRT-PCR experiments [59] is detailed in Table S2. At the end of each run, a melting curve was generated to check the specificity of the products. Relative gene expression was determined using qBasePLUS software v2.5 (Biogazelle, Zwijnaarde, Belgium). Reference genes were selected according to literature [60], and the two most stable ones were used for data normalization (Table S1).

## 3. Results

### 3.1. Cd Stress Provokes the Accumulation of (iso)Flavone Conjugates in Stem Tissue

A recent study of *M. sativa* stem tissue in response to long-term Cd exposure suggested a Cd-induced induction of the phenylpropanoid pathway [52], which diverges either into the monolignol biosynthetic pathway or flavonoid biosynthetic pathway. Monolignols are the building blocks of lignin and cell wall lignification in response to heavy metal exposure is reported in literature [61,62]. However, the cell wall of alfalfa did not show induced lignification due to Cd exposure [52].

In stem tissue of *M. sativa*, expression levels of genes involved in flavone synthesis, namely chalcone isomerase, chalcone reductase and isoflavone synthase were significantly higher in response to Cd exposure (Table 1).

**Figure 1:**
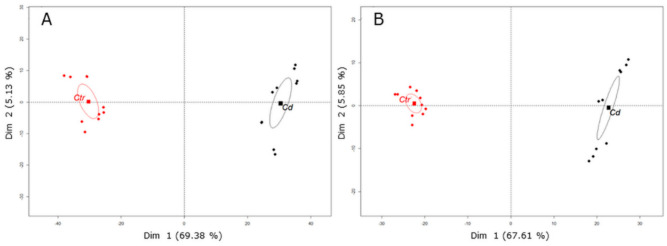
Plots of PCA revealed differences of the secondary metabolite profiles in *M. sativa* stems after long-term Cd exposure (10 mg kg^−1^ soil dry weight). Results show two distinct clusters of Cd-exposed (Cd) stem samples and control (Ctr) stem samples. Metabolites were measured by UPLC–MS in two technical replicates from five biological replicates. When samples were analyzed in (**A**) negative mode, Dim 1 and Dim 2 represent together 74.51% of the observed variance in the sample set. (**B**) In positive mode, Dim 1 and Dim 2 represent together 73.46% of the observed variance in the sample set. The barycenter of samples from control and Cd-exposed plants is indicated with a square.

To better understand the metabolic changes associated with long-term Cd exposure in *M. sativa* stems, metabolic profiling was performed using UHPLC–MS in negative and positive mode. Principal component analysis (PCA) clearly separated metabolic profiles between Cd-exposed and control stem samples (Figure 1). When samples were run in negative mode, PCA explained 74.51% of the observed data variability, thereby Dim 1 explained most of the variability (69.38%) and Dim 2 explained 5.13% (Figure 1A). In positive mode, PCA explained 73.46% of the observed data variation and Dim 1 explained most of the variability (67.61%) (Figure 1B). In negative mode, 1362 metabolite features (retention time x m z^−1^) were detected that significantly changed upon Cd exposure (with *p*-value ≤ 0.05 and fold-change ≥ 1.5) and in total 25 metabolites were putatively identified (Table 2). In positive mode, 769 metabolite features significantly changed in response to Cd (with *p*-value ≤ 0.05 and fold-change ≥ 1.5) and a total number of 26 metabolites were putatively identified (Table 3). Most identified secondary metabolites are (iso)flavones conjugated with a hexoside being hydroxylated or dihydroxylated. With only a few exceptions (N-acetyl-hexosamine derivate, tetrahydroxy(iso)flavone-hexoside, trihydroxy(iso)flavone conjugate), identified secondary metabolites accumulate in response to Cd exposure (Table 2 and Table 3). References on which identification was based are given in Table 2 and Table 3.

### 3.2. Cd Exposure Alters the Amino Acid Profile of Stems and Leaves Distinctively

**Figure 2:**
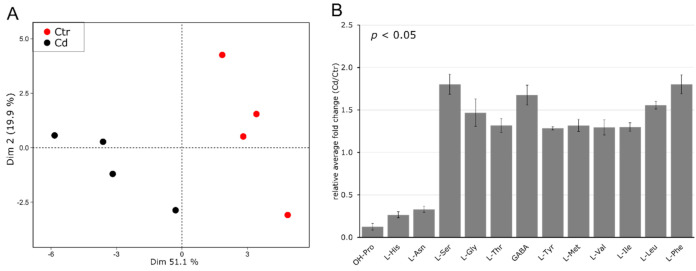
Amino acid profile in *M. sativa* stems after long-term exposure to Cd (10 mg kg^−1^ soil dry weight). Amino acids were measured using UHPLC–DAD (*n* = 4). (**A**) Principal component analysis. Dim 1 and 2 represent together 71% of the observed variance (**B**) relative fold change of significantly changed AA (±SEM). Significance was determined with a *t*-test at *p* ≤ 0.05. Absolute values for all AAs are provided in Table S3.

The effect of long-term Cd exposure on the amino acid (AA) content in *M. sativa* stems was determined using UHPLC–DAD. In order to visualize sample grouping and derived AA differences in four biological replicates of non-exposed control and Cd-exposed plants, a PCA was performed using the data of 31 quantified amino acids (Table S3). The plot shows that both conditions are clearly discriminated from each other along the first and second dimension (Figure 2A). Control plants are separated from the Cd-exposed plants along the first dimension, which explained 51.1% of the total observed variability. Significantly changed AAs in stem tissue (*p* ≤ 0.05) in response to Cd exposure are given in Figure 2B. The most abundant AA in the control samples was L-Asn (26.793 μmol g^−1^ DW) and its concentration decreased about three-fold in Cd-exposed *M. sativa* stems. Moreover, concentrations of OH-Pro (7.9-fold) and His (3.8-fold) decreased upon Cd exposure. The content of L-Ser, L-Gly, L-Thr, GABA, L-Tyr, L-Met, L-Val, L-Ile, L-Leu and L-Phe significantly increased in response to Cd exposure.

The observed AA profile in stems under Cd stress contradicts what can be found in the literature. However, most studies focus on shoots or leaves and studies focusing only on stem tissue are rather limited. Therefore, to evaluate whether this study’s used experimental conditions were comparable with those used in other studies, the AA profile of leaves was determined.

**Figure 3:**
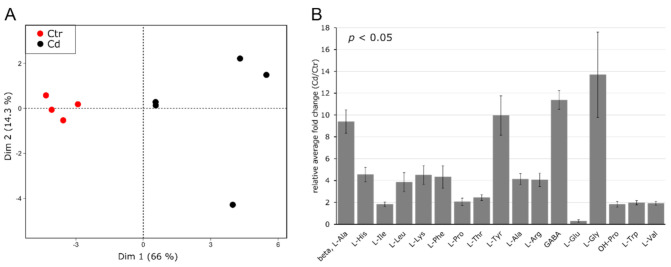
Amino acid profile in *M. sativa* leaves after long-term exposure to Cd (10 mg kg^−1^ soil dry weight). Amino acids were measured using GC–MS (*n* = 4 to 5). (**A**) Principal component analysis. Dim 1 and 2 represent together 80.3% of the observed variance (**B**) relative fold change of significantly changed AA (±SEM). Significance was determined with a *t*-test at *p* ≤ 0.05. Absolute values for all AAs are provided in Table S4.

Amino acids in leaves were identified using GC–MS, and the results clearly show a discrimination of Cd-exposed and non-exposed control plants. The first and second principal components accounted for 66.0% and 14.3% of the total variance, respectively (Figure 3A).

Out of 23 identified AAs (Table S4), 17 AAs significantly changed due to the applied stress, whereby all significantly increased in response to Cd exposure except for L-Glu, which significantly decreased (Figure 3B). Contrary to what was observed in stem tissue, L-His and OH-Pro increased 4.6- and 1.8-fold, respectively, relative to control samples. Furthermore, the abundance of L-Pro significantly increased in leaves (2.1-fold), which is in accordance with the current literature [72,73] but was not observed for stem tissue (Figure 2B, Table S3).

**Figure 4:**
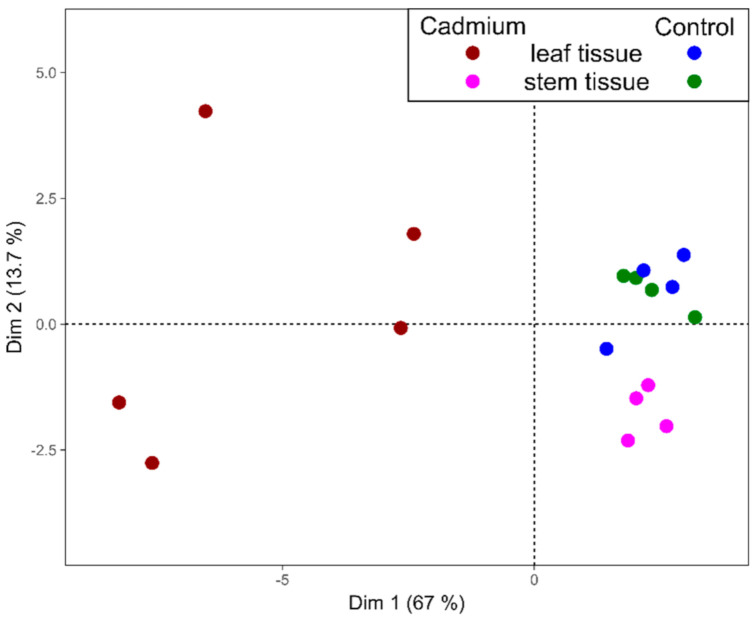
Principal component analysis demonstrates differentiation between the leaves and stems of *M. sativa* after long-term Cd exposure based on their AA profile, based on values normalized to the average of the non-exposed control for each organ separately. Dim 1 captures 67% of the variance, and Dim 2 captures 13.7% of the variance.

Under the here applied conditions, the AA profiles of both tissues are distinct, suggesting tissue-specific alterations in plant primary metabolism following long-term Cd exposure. Principal component analysis could clearly separate the AA profiles of both organs from non-exposed control and Cd-exposed plants, explaining 80.7% of the data variation in the first two dimensions (Figure 4). Stem samples exposed to Cd are only slightly distanced from the control cluster along the second dimension. However, the variance separating the sample set of Cd-exposed leaves from other sample sets is much higher, mainly along the first dimension, which indicates a strong impact of Cd on the AA profile in leaves (Figure 4).

**Figure 5:**
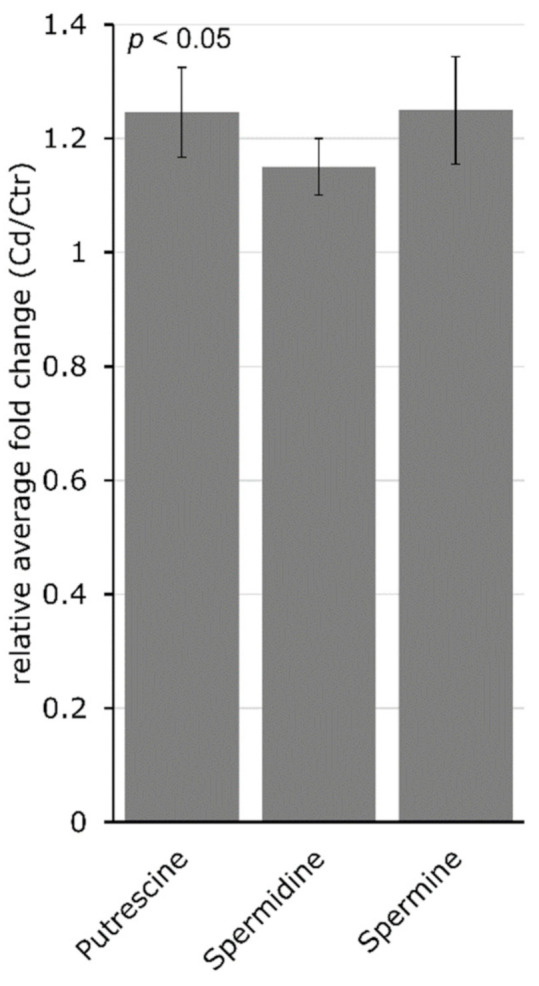
Long-term Cd exposure significantly changes the polyamine content in *M. sativa* stems. Relative average fold-change ± SEM (*n* = 5). Polyamine content was measured using HPLC-FLD. Significance of fold change was determined with a Student’s *t*-test at *p* < 0.05.

In a separate analysis, the PA content in stem tissue was determined. Long-term Cd exposure increased putrescine (Put), spermidine (Spd) and spermine (Spm) significantly (*p* = 0.04 for all) in *M. sativa* stems. Putrescine increased about 1.2-fold, Spd about 1.1-fold and Spm about 1.2-fold (Figure 5). Targeted genes involved in PA synthesis showed an increased expression upon Cd exposure. Among these, the changes in the expression of arginine decarboxylase (ADC) and S-adenosylmethionine decarboxylase (SAM DC) were insignificant. In addition, diamine oxidase (DAO) had a significantly decreased expression in Cd-exposed *M. sativa* stems (Table 1).

### 3.3. Glutathione Plays a Minor Role during Long-Term Cd Exposure

In *M. sativa* stem tissue, long-term Cd exposure stimulated the expression of genes involved in the biosynthesis of GSH, such as homoglutathione synthase and glutathione synthase 2 (Table 1). However, the expression levels of genes involved in the turnover between oxidized and reduced GSH did not show significant changes in response to long-term Cd exposure (glutathione reductase 1 and 2) with the exception of monodehydroascorbate reductase, for which a significant increased expression was determined. No significant changes in total GSH (control: 400.74 nmol g^−1^ FW; Cd: 389.30 nmol g^−1^ FW) nor in reduced GSH (control: 284.58 nmol g^−1^ FW; Cd: 273.52 nmol g^−1^ FW) were observed in response to Cd exposure. In addition, the measured average concentration of the oxidized form GSSG remained the same in non-exposed control (58.08 nmol g^−1^ FW) and Cd-exposed *M. sativa* stems (57.89 nmol g^−1^ FW).

## 4. Discussion

The objective of this study was to determine the effects of long-term Cd exposure (10 mg kg^−1^ soil DW) on the profile of primary and secondary metabolites in *M. sativa* by focusing on stem tissue. Plant growth was negatively influenced by Cd exposure in the juvenile plant stage but plants phenotypically recovered when they were more mature and consequently no difference in the total biomass produced was observed at the end of the experiment [50]. In agreement with these results, previous studies undertaken in rice, *M. sativa* and *Trifolium repens* L. reported a marked impact of heavy metal exposure during germination and early plant growth phases, while minimal effects were observed in later growth stages [74,75,76]. This observation might result from the establishment of a new steady state, which permits acclimation and allows *M. sativa* to grow under Cd stress. Despite GSH being a key player in the defense against Cd and a crucial antioxidant, it seems to only play a minor role in stem tissue of *M. sativa* after long-term exposure to Cd. Although the expression of genes related to GSH synthesis was induced due to the applied stress (*homoglutathione synthase* and *glutathione synthase 2* (Table 1)), suggesting that plants produce more GSH, no changes between non-exposed control and Cd-exposed *M. sativa* stems were observed in the total GSH content, reduced GSH content as well as average concentration of the oxidized form GSSG. Changes in glutathione concentrations and redox state often occur rapidly after the start of Cd exposure and are then restored to control levels [77]. It is likely that changes in GSH metabolism and the redox cycle do occur in Medicago stems but disappear during long-term exposure and it was demonstrated in earlier studies that GSH synthesis often does not correlate with plant stress tolerance [78,79]. Other compounds and/or enzymatic activities might be of higher importance to establishing a new metabolic homeostasis and facilitate acclimation to long-term Cd exposure.

By studying the metabolic profile, further insights can be gained about the key processes and molecules involved in such acclimation.

### 4.1. Cd Increases the Abundance of (iso)Flavones

Flavonoids and (iso)flavonoids derive from the phenylpropanoid pathway, which is shared with the monolignol biosynthetic pathway and lignin synthesis. Stress stimulates the phenylpropanoid pathway [80] and results in increased lignification of the cell wall [81,82]. However, when the lignin content was analyzed in stems of *M. sativa* after long-term Cd exposure, neither changes in the content nor in the lignin composition were observed [52]. Nonetheless, the expression of the genes *C4H* and *4CL* significantly increased and an increasing trend in the expression of *PAL* was detected upon Cd exposure [52], suggesting an induction of the phenylpropanoid pathway in response to Cd. It is, however, noteworthy that the activity of PAL does not necessarily correlate with the accumulation of its RNA transcript. Moreover, the activity is clearly driven by the availability of its substrate Phe [83], and an increased Phe level in the stems of Cd-exposed *M. sativa* is shown here by two independent methods (Table 2, Figure 2B). Furthermore, ethylene biosynthesis was stimulated by Cd exposure in *M. sativa* stems [52], which would promote PAL activity [84,85] and enhances the accumulation of phenolic compounds [86].

Secondary metabolites in stem tissue of *M. sativa* were determined with UPLC–MS. The results showed an accumulation of (iso)flavones in response to Cd. Those (iso)flavones were glyco-conjugated and/or hydroxylated (Table 2 and Table 3), and the results are in accordance with (iso)flavone glycoconjugate accumulation observed upon Cd exposure in roots of yellow lupine (*Lupinus luteus*) [87]. In nature, flavonoids and (iso)flavonoids are glyco-conjugated [64] as it increases the solubility as well as the stability of (iso)flavones, promotes their uptake into the vacuole, facilitates their transport through membranes [88] and can be crucial for their bioactivities [89]. Cumulative evidence suggests that the chemical structure of (iso)flavones determines their bioactivity and antioxidative efficiency [90,91]. Hydroxylation of flavones increases their antioxidative properties and capacity to complex with metal cations, which is co-ordinated by the hydroxyl group. Therefore, the ability to form such metal complexes strongly depends on the number and position of the hydroxyl group [92,93]. Identified (iso)flavones in *M. sativa* stems have glycosylations and/or hydroxylations (Table 2 and Table 3), effectively contributing to their function to scavenge ROS, preventing oxidative stress and furthermore facilitating metal chelation. Such metal complexes are stored in the vacuole for detoxification [94] and it is thought that glyco-conjugation might be essential in this process given that it promotes vacuolar uptake.

The observed accumulation of (iso)flavones was accompanied by an increased expression of genes of their biosynthetic pathway (Table 1), suggesting an enhancement of the phenylpropanoid pathway in favor of secondary metabolite synthesis rather than lignin synthesis. This is in accordance with previous observations made in lupine seedlings under Cd stress [34,87].

### 4.2. Cadmium Induces Changes in the Abundance of Amino Acids and Amino-Acid-Derived Molecules

Changes in the AA profile constitute an important mechanism during plant adaptation to Cd stress [30], and higher tolerance to Cd and elevated Cd accumulation was characterized through an increased accumulation of AAs [95]. Previous studies assigned an important role in Cd stress adaptation to the AAs Met, His and Trp [96] as well as Pro [73,97]. Asparagine, His and Pro can form complexes with metal ions and thus contribute to detoxification [98,99,100] and confer tolerance to the plant. Exogenously applied, Pro alleviates stress symptoms and maintains nutrient uptake [101]. Furthermore, Pro possesses ROS scavenging potential by directly reacting with H_2_O_2_ and •OH to form stable radical adducts of proline and hydroxyproline [102], thereby reducing ROS levels [103]. In contradiction with most literature, a significant decrease in L-Asn and L-His of about 3-fold and 3.8-fold, respectively (Figure 2B), was observed in stems of *M. sativa* after long-term Cd exposure. Yet, Asn was the most abundant AA in *M. sativa* stems (Ctr: 26.79 μmol g^−1^ DW; Cd: 8.83 μmol g^−1^ DW), which is the dominant amino acid in the xylem sap of *M. sativa*, representing the primary transport form of nitrogen [104]. Its decrease can be a reflection of an interference with nitrogen fixation caused by Cd exposure [105]. A decreasing trend was also observed for Pro (Ctr: 9.46 μmol g^−1^ DW; Cd: 6.07 μmol g^−1^ DW; *p*-value = 0.152; Table S3). Despite being an important AA during plant stress exposure [106,107,108], previous studies reported a decrease in Pro levels over time after long-term Cd exposure [31,109]. Although the observations made are quite surprising considering the importance of those AAs during metal stress, it should be considered that roots are the main organ of Cd accumulation in *M. sativa* after long-term Cd exposure [50]. Detoxification of Cd is primarily important at the root-cell level rather than in stems and an elevated requirement of complexing molecules such as Asn and His in roots can be assumed.

This study focused on stem tissue of *M. sativa* but most available data about AA accumulation in response to heavy metal stress were obtained in entire shoots or leaves. Therefore, the AA profile was also determined in leaves of *M. sativa* in order to compare both data sets in the background of recent literature and to evaluate whether the results of the experimental set-up we used are comparable to those of other studies. The quantification of AAs in *M. sativa* leaves revealed higher contents of Asn and His upon long-term Cd exposure as well as the accumulation of Pro, which was accompanied by an increased tyramine content (Figure 3B). Tyramine is an aromatic monoamine that is believed to be involved in the regulation of Pro accumulation under stress conditions [110] and was reported to accumulate in response to Cd stress [111].

Leaves and stems of *M. sativa* showed distinct AA profiles after long-term exposure to Cd, and a PCA could clearly separate AA profiles of samples from the non-exposed control and Cd-exposed plants (Figure 4), whereby leaf samples had the highest variance in their AA profile due to Cd exposure. It is important to mention that leaves are the metabolically most active organ, and the impact of unfavorable conditions such as Cd stress therefore might have much stronger effects, which is illustrated by the PCA (Figure 4). A different AA profile in different organs can play a significant role during adaptation to Cd stress [96]. A study on two related Asteraceae species revealed distinctive AA profiles in roots, stems and leaves. While the total content of free AAs was highest in the stems in the hyperaccumulating species, free AAs strongly accumulated in the leaves of the non-tolerant species exposed to Cd [95], suggesting a different strategy in the defense response to Cd. In this study, the content of OH-Pro, an oxidation product of Pro and indicator for oxidative stress levels, increased in leaves due to Cd exposure (Figure 3B) but decreased about 8-fold in *M. sativa* stems (Figure 2B), suggesting a reduced oxidative stress level in stems, which is coherent with an unaltered GSH metabolism described in this study. This indicates differently altered oxidative stress levels in leaves and stems in the Cd-exposed plants compared to the control plants. *M. sativa* plants accumulated Asn, His and Pro in leaves rather than in stems. The leaf tissue consists of many metabolically very active cells, while this is lower in cells from the stems. Asparagine, His and Pro have a high potential for metal chelation and ROS scavenging, and their accumulation in leaves might help to protect and maintain photosynthesis. The protection of stem tissue probably depends on different mechanisms than that of leaves, and long-term Cd exposure significantly increased the content of the Pas Put, Spd and Spm (Figure 5) in *M. sativa* stems as well as the expression levels of the genes SpdS and SpmS that are involved in their biosynthesis (Table 1). Polyamines are involved in plant responses to multiple biotic and abiotic stresses [112] such as Cd exposure [113], showing a concentration- and time-dependent accumulation [114]. Polyamines alleviate the toxic effects of Cd [115], decrease the production of ROS, prevent lipid peroxidation [116] and permit stress tolerance. Moreover, PAs are important for cell membrane and cell wall stabilization [117], and therefore their accumulation in stems may help to maintain cell integrity during Cd exposure.

### 4.3. Abscisic Acid Supports Tolerance Acquisition to Cd

Like ethylene, abscisic acid (ABA) is often considered a plant stress hormone functioning as a mediator and major internal signal during abiotic stress [118]. Both hormonal signals are integrated during plant stress responses [119]. Not only did long-term Cd exposure stimulate ethylene biosynthesis in *M. sativa* stems [52] but it also provoked elevated levels of ABA in the same tissue (Table 2). Abscisic acid stimulates the production of ethylene resulting in the accumulation of phenolic compounds (Table 2 and Table 3) via ethylene-mediated PAL activation [84].

When applied during Cd stress, ABA reduces the plant transpiration rate and increases Cd tolerance [120]. Transpiration is the motor for Cd translocation from roots to shoots [121]. Low transpiration enforces Cd to accumulate in the roots, thereby lowering the concentration in the aerial parts, as was observed in *M. sativa* upon long-term Cd exposure [50]. This seems to be an important mechanism during tolerance acquisition. Furthermore, ABA accumulation is initiated by elevated ROS levels. ROS homeostasis is modulated by the plant’s flavone content, which in turn is influenced by ethylene [122]. During long-term Cd exposure, *M. sativa* was only impacted by the stress at its juvenile stage and recovered during plant maturation [50], which implies tolerance acquisition to Cd. Reasonably, it can be assumed that acclimation of *M. sativa* during long-term Cd exposure must involve a tightly regulated interplay between ROS production, the antioxidant activity of isoflavones as well as ethylene and ABA signaling.

## 5. Conclusions

A combined approach of targeted and untargeted metabolic analysis was used to determine the effect of long-term Cd exposure on primary and secondary metabolites in *M. sativa* stems. Control and Cd-exposed plants can be discriminated by their AA pattern, whereby differences in the accumulation of several AA appeared between stems and leaves, suggesting a different strategy in the defense response to Cd in both organs, which in general differ in their metabolic activity. Furthermore, long-term Cd exposure stimulated the phenylpropanoid pathway in favor of flavonoid/isoflavonoid synthesis rather than monolignol biosynthesis and lignin formation. Flavonoids and (iso)flavones have a designated function in ROS scavenging, thereby reducing oxidative stress and cell damage. However, the important antioxidant GSH plays a minor role during long-term Cd exposure. However, the decreased accumulation of 4-hydroxyproline in stems of long-term exposed plants suggests that other mechanisms (synthesis and accumulation of metabolites and/or enzymatic antioxidant activities) are sufficient to restore the redox balance. Our results emphasize the importance of primary and secondary metabolites for acclimation potential during long-term Cd exposure. The establishment of a new metabolic homeostasis enables *M. sativa* to perform well under inept conditions as no differences in phenotype or biomass production were observed.

## Figures and Tables

**Table 1:** Changes of normalized relative gene expression quantities in *M. sativa* stems upon cadmium (Cd) exposure. Normalized expression values are given relative to the control set at 1.00. Values are given as an average of 5 replicates. A *t*-test was performed to determine the significance (*p* ≤ 0.05). Green significantly up-regulated; red significantly down-regulated.

Gene Annotation	Contig ID *M. sativa*	Rel. Norm. Gene Expression	*p*-Value
Polyamine synthesis			
*arginine decarboxylase*	46,745	1.123	0.548
*diamine oxidase*	14,769	0.800	0.042
*glutamate decarboxylase*	103,359	1.685	0.019
*SAM decarboxylase*	9152	1.141	0.459
*spermidine synthase*	5341	1.213	0.042
*spermine synthase*	92,526	1.341	0.032
Flavone synthesis			
*chalcone isomerase*	60,809	1.809	0.019
*chalcone reductase*	59,796	2.753	0.019
*chalcone synthase*	99,463	1.247	0.371
*isoflavone synthase*	10,252	2.699	0.019
Glutathione synthesis and redox cycle			
*homoglutathione synthetase*	[63]	1.355	0.01
*γ-glutamylcysteine synthetase*	[63]	0.959	0.361
*glutathione reductase 1*	[63]	1.105	0.071
*glutathione reductase 2*	[63]	1.043	0.681
*monodehydroascorbate reductase*	[63]	1.177	0.048
*glutathione synthetase 2*	10,444	1.384	0.004

**Table 2:** List of differentially abundant compounds in *M. sativa* stems after long-term Cd exposure. Data were obtained by UHPLC–MS in negative mode, with MS/MS experiments. The relative abundance of each peak in stems of control and Cd-exposed plants is based on the selected ion current and is presented as the average normalized value of two technical replicates from five biological replicates. Rt, retention time; nd, not detected.

R_t (min)_	Relative Abundance		Fold	[M − H]	Error (ppm)	Molecular Formula	MSMS [M − H]	Putative Identity	Reference
	**Cadmium-Exposed**	**Control**							
2.23	446	281	1.59	164.0719	1.22	C_9_H_11_NO_2_	147.0434 103.0539- 164.0693 72.0108	L-Phe *	
2.50	1	89	89.00	274.0932 [M − H_2_O − H]	−0.11	C_11_H_19_NO_8_	89.0280 274.0929 100.0413 159.8914 232.0821	*N*-acetyl-hexosamine derivative	
15.37	21	1	39.40	669.1687 [M + FA − H]	2.18	C_28_H_32_O_16_	299.0537 284.0294 461.1077	Trihydroxy-methoxy (iso)flavone di-hexoside	[64,65,66]
18.18	284	30	9.36	299.0768	−1.47	C_13_H_16_O_8_	137.0227 109.0266 135.0047 153.0146 91.0181	Hydroxybenzoyl-hexoside	[67]
19.07	464	69	6.69	605.1163	2.50	C_27_H_26_O_16_	113.0234 253.0478 351.0579 85.0294 193.0316	Dihydroxy(iso)flavone di-hexuronide	[64,65]
19.41	21	1	24.86	299.0773	0.20	C_13_H_16_O_8_	137.0198 135.0049 100.9291	Hydroxybenzoyl-hexoside	[65,67]
19.49	171	15	11.37	605.1164	2.63	C_27_H_26_O_16_	113.0211 253.0500 85.0285 351.0572 193.0314	Dihydroxy(iso)flavone di-hexuronide	[64,65]
19.64	63	6	10.26	461.1093	0.78	C_22_H_22_O_11_	284.0273 299.0545 255.0303 135.0046	Trihydroxy methoxy-(iso)flavone hexoside	[64,65,66]
20.06	228	50	4.58	429.0824	−0.75	C_21_H_18_O_10_	253.0489 85.0297 113.0224 135.0058 117.0322	Dihydroxy(iso)flavone-hexuronide	[64,65]
20.41	50	7	6.88	429.0823	−0.98	C_21_H_18_O_10_	253.0481 117.0344 135.0079 85.0281 280.7946	Dihydroxy(iso)flavone-hexuronide	[64,65]
21.83	52	123	2.35	447.0931	−0.42	C_21_H_20_O_11_	284.0327 447.1017	Tetrahydroxy(iso)flavone-hexoside	[64,68]
23.22	26	nd	treated only	283.0610	−0.71	C_16_H_12_O_5_	268.0387 147.9081 211.0346 283.0632	Dihydroxy- methoxy(iso)flavone	[64,66,69]
23.80	59	2	33.23	283.0608	−1.41	C_16_H_12_O_5_	268.0340 211.0382 239.0319 195.0406	Dihydroxy-methoxy(iso)flavone	[65,70]
23.96	68	10	6.59	253.0504	−0.91	C_15_H_10_O_4_	253.0470 117.0329 135.0080 91.0203 133.0290	Dihydroxy(iso)flavone	[65]
24.03	28	1	19.01	313.0712	−1.79	C_17_H_14_O_6_	255.0286 298.0491 283.0201 227.0312 171.0404	Dihydroxy- dimethoxy-(iso)flavone	[65]
25.11	151	68	2.22	263.1285	−1.44	C_15_H_20_O_4_	153.0898 219.1360 204.1122 136.0514	Abscisic acid*	
26.22	29	2	18.56	267.0663	0.07	C_16_H_12_O_4_	252.0402 223.0395 195.0422 267.0628 251.0305	Hydroxy-methoxy(iso)flavone	[64,70]
26.8	588	88	6.69	267.0664	0.45	C_16_H_12_O_4_	252.0406 223.0371 195.0419	Hydroxy-methoxy(iso)flavone	
27.73	39	nd	treated only	299.0920	−1.62	C_17_H_16_O_5_	135.0072 91.0170 299.0911 284.0303 269.0444 256.0339	Unknown compound	
28.34	129	17	7.65	473.1464 [M + FA **−** H]	2.28	C_24_H_26_O_10_	254.0571 209.0582 211.0394 225.0548 269.0838	Hydroxy-methoxy-pterocarpan malonate hexoside derivative	[71]
30.19	12	1	10.12	297.0763	−1.85	C_17_H_14_O_5_	239.0363 282.0544 183.0049 195.0418 254.0552 211.0394	Dimethoxy-hydroxy(iso)flavone	[64]
34.76	38	3	14.57	313.0715	−0.83	C_17_H_14_O_6_	255.0277 298.0473 227.0329 270.0526 183.0420	Dihydroxy- dimethoxy(iso)flavone	[64,65]
36.49	71	3	22.64	509.1244	0.41	C_30_H_22_O_8_	237.0909 263.0704 135.0058 373.1054 399.0872	Hydroxylated chalcone dimer	[65]

* Identity of the compound was confirmed with an authentic standard.

**Table 3:** List of differentially abundant compounds in *M. sativa* stems after long-term Cd exposure. Data were obtained by UHPLC–MS in positive mode, with MS/MS experiments. The relative abundance of each peak in stems of control and Cd-exposed plants is based on the selected ion current and is presented as the average normalized value of two technical replicates from five biological replicates. Abbreviations: Rt, retention time; nd, not detected.

R_t (min)_	Relative Abundance		Fold	[M + H]	Error (ppm)	Molecular Formula	MSMS [M + H]	Putative Identity	Reference
	**Cadmium-Exposed**	**Control**							
2.5	1	114	113.29	276.107	−2.82	C_11_H_19_NO_8_	138.0521 96.0424 144.0625 126.0524 84.0413	*N*-acetyl-hexosamine derivative	
16.62	82	312	3.92	271.0593	−2.95	C_15_H_10_O_5_	215.0675 149.0229 57.0700 43.0562	Trihydroxy(iso)flavone conjugated	[64,65]
18.17	288	16	17.84	241.0853	−2.57	C_15_H_12_O_3_	107.0438 131.0451 147.0454 123.0414 77.0363	Unknown compound	
19.04	380	65	5.83	607.1284	−1.58	C_27_H_26_O_16_	255.0602	Dihydroxy(iso)flavone-di-hexuronide	[64,65]
19.46	139	15	9.17	607.01278	−2.57	C_27_H_26_O_16_	255.0560	Dihydroxy(iso)flavone di-hexuronide	[64,65]
19.65	36	3	12.82	463.1224	−2.35	C_22_H_22_O_11_	301.0698 241.0524 269.0445 213.0523 145.0240	Trihydroxy-methoxy-(iso)flavone hexoside	[64,65,66]
20.07	281	70	3.99	431.0957	−3.64	C_21_H_18_O_10_	255.0646 137.0214 145.0236 119.0462	Dihydroxy(iso)flavone hexuronide	[64,65]
20.37	58	10	6.03	431.0959	−3.18	C_21_H_18_O_10_	255.0629 137.0224 145.0267 119.0485	Dihydroxy(iso)flavone hexuronide	[64,65]
21.13	76	nd	treated only	489.1382	−1.9	C_24_H_26_O_12_	241.0828 131.0497 147.0428 213.0866 107.0483	Unknown compound	[65]
21.82	24	53	2.22	449.1063	−3.43	C_21_H_20_O_11_	287.0529 153.0133 269.0445 259.0586	Tetrahydroxy(iso)flavone-hexoside	[64,68]
23.07	104	4	23.42	563.1382	−2.36	C_26_H_26_O_14_	315.0839 299.0534 254.0531	Dihydroxy- dimethoxy-(iso)flavone malonate-hexoside derivative	[64,65]
23.22	70	1	61.76	533.1279	−2.01	C_25_H_24_O_13_	285.0756 270.0515 253.0510 225.0545 242.0553	Dihydroxy methoxy-(iso)flavone malonate-hexoside derivative	[64,65]
23.94	61	17	3.51	255.0640	−4.67	C_15_H_10_O_4_	255.0638 137.0209 145.0263 119.0462 93.0307	Dihydroxy(iso)flavone	[65]
24.02	86	2	37.18	563.1376	−3.43	C_26_H_26_O_14_	315.0834 300.0593 283.0562 255.0645	Dihydroxy- dimethoxy-(iso)flavone malonate-hexoside derivative	[64,65]
24.78	70	1	90.39	563.1376	−3.43	C_26_H_26_O_14_	315.0868 207.0628 175.0371 300.0625 147.0419	Dihydroxy- dimethoxy-(iso)flavone malonate-hexoside derivative	[64,65]
25.19	32	9	3.75	299.0907	−2.34	C_17_H_14_O_5_	299.0955 284.0663 93.0674 256.0754 166.0265	Dimethoxy-hydroxy(iso)flavone	[64]
26.18	74	7	9.93	517.1324	−3.19	C_25_H_24_O_12_	269.0809 254.0608 237.0551 213.0890 107.0488	Hydroxy-methoxy(iso)flavone malonyl hexose	[64,69,71]
26.79	1999	287	6.97	517.1324	−3.19	C_25_H_24_O_12_	269.0804 254.0569 213.0902 237.0544 226.0615	Hydroxy-methoxy-(iso)flavone malonate-hexoside derivative	[64,71]
26.90	593	50	11.85	547.1433	−2.41	C_26_H_26_O_13_	299.0914 284.0680 256.0706 239.0703	Dimethoxy-hydroxy(iso)flavone malonate-hexoside derivative	[64]
27.55	46	nd	treated only	533.1272	−3.26	C_25_H_24_O_13_	151.0376 123.0401 285.0806	Unknown compound	
28.35	611	100	6.10	519.1478	−3.70	C_25_H_26_O_12_	137.0575 271.0961 161.0581 123.0421 201.1603	Hydroxy methoxy-pterocarpan malonate-hexoside derivative	[64,69,71]
28.50	32	nd	treated only	329.1011	−2.70	C_18_H_16_O_6_	329.1008 313.0722 285.0746 295.0591 267.0649	Unknown compound	
30.18	387	105	3.69	299.0904	−3.34	C_17_H_14_O_5_	299.0915 284.0663 256.0721 132.0539 227.0699	Dimethoxy-hydroxy(iso)flavone	[64,65]
31.13	73	19	3.86	271.0954	−4.02	C_16_H_14_O_4_	137.0573 109.0621 79.0524 123.0413 161.0578	Pterocarpan	[64,69]
36.48	23	nd	treated-only	511.1372	−3.01	C_30_H_22_O_8_	137.0214 375.1088 439.2446 265.0972	Hydroxylated chalcone dimer	[65]

## Supplementary Materials

The following are available online at https://www.mdpi.com/2073-4409/9/12/2707/s1, Table S1: Forward (Fwd) and reverse (Rev) primers used to determine gene expression levels via quantitative real-time PCR. The Alfalfa Gene Index and Expression Atlas Database was used to obtain the coding sequences for the genes of interest which are given as contig numbers. Table S2: Quantitative real-time PCR parameters according to the Minimum Information for publication of Quantitative real-time PCR Experiments (MIQE) guidelines derived from Bustin et al. (2009) [59]. All procedures were performed according to the manufacturer’s protocols. Table S3: Amino acids in stem tissue of *M. sativa* after long-term Cd exposure. Table S4: Relative content of primary metabolites in the leaves of *M. sativa* after long-term Cd exposure.
