# Supplementary material for: Long-Term Cd Exposure Alters the Metabolite Profile in Stem Tissue of Medicago sativa
Source: Cells. 2020 Dec 17;9(12):2707. doi: 10.3390/cells9122707 (PMC7765984; doi:10.3390/cells9122707)
Supplement: Supplementary file 1 [file cells-09-02707-s001.zip › Table S1 and S2.pdf]

Table S1: Forward (Fwd) and reverse (Rev) primers used to determine gene expression levels via quantitative real-time PCR. The Alfalfa Gene Index and Expression Atlas Database was used to obtain the coding sequences for the genes of interest which are given as contig numbers.

Gene	Contig ID	Sequence (5´→ 3´)	Amplicon length (bp)	Efficiency (%)
Polyamine synthesis				
arginine decarboxylase	46745	Fwd: GCTTGAATCTCTTCACGCC<br>Rev: TCCTCCACAACAAACCTATCC	114	109
diamine oxidase	14769	Fwd: CTGGTGGTCAGAATGATTGC<br>Rev: ACCCCAACTTTTATGCTGC	89	102
glutamate decarboxylase	103359	Fwd: ATGGATGAATACCCCGTCAC<br>Rev: AACAGTGCCAACACCAACTG	114	107
SAM decarboxylase	9152	Fwd: CAAGCAAGTCGTTTGAGCAG<br>Rev: ACAGAACCACCCATTCCAAG	94	101
spermidine synthase	5341	Fwd: ACCGACATTCACACCAGAC<br>Rev: TCTCCTTCTCCAAACCATTCC	137	107
spermine synthase	92526	Fwd: TCTTTTATGTCATCCAAACCCC<br>Rev: AATCCACCACCTCCTTGTC	134	108
Flavonoide synthesis				
chalcone isomerase	60809	Fwd: CAAGGCAGTTTCATCAGCAG<br>Rev: AAGCACCCTCGTTCAACAAC	113	108
chalcone reductase	59796	Fwd: TCCTGCTCTTCAAAAATCTCTC<br>Rev: TCCCAAACACCTTTCACATC	150	108
chalcone synthase	99463	Fwd: ACCTTCATTGGATGCTAGGC<br>Rev: GTGGTGCAAACGATCAAGTG	132	108
isoflavone synthase	10252	Fwd: TGCTATGGTTCCCTTTGGAC<br>Rev: GATTTCTCGGCTCCTCAATG	112	102
Glutathione synthesis and redox cycle				
homoglutathione synthetase	Cui et al. 2012	Fwd: GGCACCAAAAAGATTCAACA<br>Rev: AACTCCACAACCCTGCAAAG		122
γ-glutamylcysteine synthetase	Cui et al. 2012	Fwd: CATGGAAAGTGGGAGCAATC<br>Rev: AGCAAGAAGCCGAATACACC		102
glutathione reductase 1	Cui et al. 2012	Fwd: AGGACGGTGAACCTGATTTG<br>Rev: GAATCTAGCAGCACGAACAC		99

glutathione reductase 2	Cui et al. 2012	Fwd: TTCCGTTCTCCACAATCTCAT<br>Rev: TTCACTGTCATATCTCCATCCAA	90
monodehydroascorbate reductase	Cui et al. 2012	Fwd: TCAAAGGGCAGGTTGAAGAG<br>Rev: AAGGGAACGTAGCAACATCG	102
glutathione synthetase 2	10444	Fwd: AACGATGGCAGAAGTTGACC<br>Rev: AGCCAGCCCTGAAGTAAATG	102
Reference genes			
cyclophilin	Guerriero et al., 2014	Fwd: CAAACTTTCCTGACGAGTCACC<br>Rev: ACGGTCAGCAATTGCCATTG	104
eIF4A	Guerriero et al., 2014	Fwd: TGCTAAGTTGCCTGAAACCG<br>Rev: TGCCCATGTTTTCACCTTGC	96

Table S2: Quantitative real-time PCR parameters according to the Minimum Information for publication of Quantitative real-time PCR Experiments (MIQE) guidelines derived from Bustin *et al*. (2009). All procedures were performed according to the manufacturer's protocols.

**Sample/Template**	
Source	Stems of *Medicago sativa* plants cultivated in soil
Method of preservation	Liquid nitrogen
Storage time	12 month at -80 °C
Handling	Frozen
Extraction method	Phenol-free Total RNA isolation: RNAqueous™ Kit (Life Technologies)
RNA: DNA-free	TURBO DNA-free™ Kit (Life Technologies)<br>Design of intron-spanning primers whenever possible
Concentration	NanoDrop®: ND-1000 Spectrophotometer (Thermo Fischer Scientific)
**Assay optimisation and validation**	
Accession number	Table S1
Amplicon details	Amplicon size: Table S1
Primer sequences	Table S1
In silico	Primer design with the Primer3Plus online tool (www.bioinformatics.nl/cgi-bin/primer3plus/primer3plus.cgi) and primer analysis with OligoAnalyzer 3.1 (https://eu.idtdna.com/calc/analyzer)
Empirical	A primer concentration of 100 nM was used<br>Annealing temperature: 60°C
Priming conditions	Combination of oligodT-primers and random hexamers
PCR efficiency	Dilution series (slope, y-intercept and r^2; Table S1)
Linear dynamic range	Samples are situated within the range of the efficiency curve
**Reverse transcription - PCR**	
Protocols	As stated in the materials and methods section
Reagents	As stated in the materials and methods section
No template control (NTC)	Cq and dissociation curve verification
**Data analysis**	

Specialist software	ViiA7 Real-Time PCR system (Applied Biosystems)<br>qBase^{PLUS} software v2.5 (Biogazelle)
Statistical justification	Five biological replicates<br>Relative expression of each gene was calculated as an average after elimination of outliers<br>Log transformation of the data<br>Significance of changes in gene expression was determined automatically qBase^{PLUS} software v2.5 (Biogazelle)
Normalisation	Two reference genes were selected as described in the materials and methods section
